# Parental Perceptions and Concerns Regarding Infection Control in a Pediatric Dental Setting Post Covid-19 Pandemic in Saudi Arabia: A Cross-Sectional Study

**DOI:** 10.3290/j.ohpd.c_2693

**Published:** 2026-05-28

**Authors:** Basim Almulhim

**Affiliations:** a Basim Almulhim Associate Professor, Department of Orthodontics and Pediatric Dentistry, College of Dentistry, Qassim University, Buraydah, Saudi Arabia. Conceived the study idea, collected and analyzed the data, wrote the manuscript.

**Keywords:** children, infection control, parental concerns, pediatric dentist.

## Abstract

**Purpose:**

To evaluate parental perceptions and concerns regarding post-COVID-19 pediatric dental infection control measures in Saudi Arabia.

**Materials and Methods:**

This study applied a questionnaire-based cross-sectional analysis. The questionnaire consisted of demographic characteristics and six closed-ended questions. The resultant data were entered and analyzed utilizing SPSS software. Descriptive analysis utilizing frequency and percentage was conducted for qualitative data, and a chi-squared test was performed. A p-value below 0.05 was considered statistically significant.

**Results:**

A total of 400 responses were available for the final analysis. The majority of the parental participants (43.2%) were 30 to 39 years old; 51.2% of the study population were male, and the remaining 48.8% of participants were female. The majority of parents felt that wearing gloves (97.8%; 95% CI = 96.3, 99.2), a dental mask (98%; 95% CI = 96.6, 99.4), and protective eyewear (89.5%; 95% CI = 86.5, 92.5) while treating their child was essential. The majority of parents considered it necessary to change masks (76.3%; 95% CI = 72.1, 80.4) and clean eyewear (79.0%; 95% CI = 75.0, 83.0) after treating every patient. Almost 50.5% (95% CI = 45.6, 55.4) of parents expressed no concern about contracting an infection at a pediatric dentist’s office. A significant difference was noted among several questions based on gender and levels of education.

**Conclusion:**

The majority of parents reported positive perceptions, and there was an absence of concern regarding the risk of infection during dental visits. However, to guarantee compliance with infection control protocols in pediatric dental settings, additional efforts are required to provide parents with precise information and a comprehensive understanding of the relevant guidelines.

In the dental setting, providing treatment while ensuring adherence to infection control guidelines plays a vital role in ensuring the safety of both patients and healthcare providers. Frequently, the risk of infections is correlated with some dental procedures, whether they are related to diagnostic, clinical, or therapeutic procedures, which is a significant contributor to mortality and morbidity.^13^ The close proximity between patient and dentist during dental treatment can increase the likelihood of cross-infection via aerosols. If the appropriate personal protective equipment (PPE) is not worn and the necessary precautions are ignored, the risk of infection transmission during dental treatment increases. Additionally, the risk of patient-to-patient cross-contamination and disease transmission has led to mounting concerns about infection control.^23^ Typically, infection with different pathogens may remain asymptomatic in the patient’s oral cavity or respiratory tract, including HIV/AIDS, hepatitis viruses, cytomegaloviruses, herpes simplex viruses, mycobacterium tuberculosis, or any microorganisms, and the risk of infection transmitted during dental settings is high through air droplets, saliva, blood, or contaminated dental instruments.^4^ Additionally, the transmission of blood-borne infections is not uncommon.^15^ Furthermore, identifying infections during asymptomatic periods or incubation periods is challenging.^22^ Globally, during pandemics of communicable diseases such as COVID-19, dental clinics face a significant risk of disease transmission and must strictly adhere to infection control protocols. The aforementioned points emphasize the importance of infection control in dental settings, as one study has demonstrated that improper adherence to infection control protocols can result in the detection of numerous microorganisms in dental offices.^23^ The pediatric patient is more susceptible to infection than an adult and significantly differs.^25^ Zingg et al^26^ identified that the most common types of infection in children are bloodstream infections, followed by lower respiratory tract infections. The human papillomavirus (HPV) – the most common sexually transmitted infection in children – is linked to most cases of anogenital and oropharyngeal neoplasia.^21^ Stull et al^21^ advocated that ongoing dental education can enhance practitioners’ knowledge, comfort, and confidence in addressing HPV with parents; therefore, education, awareness, and training directed towards parents and guardians are of the utmost importance. Ibrahim et al^10^ assessed the infection control knowledge, attitude, and practices of patients attending dental clinics in Saudi Arabia and reported positive scores for attitude; however, they reported that the scores for knowledge and practice could be improved via the introduction of educational programs. Pediatric patients may not necessarily be aware of infection control protocols and the risks associated with dental procedures; therefore, parents play a pivotal role in enhancing patient safety in pediatric dental healthcare settings.^9^ No previous Saudi study has focused entirely on parental perceptions and concerns regarding infection control in pediatric dental settings post the COVID-19 pandemic. Therefore, this study was designed to evaluate parental perceptions and concerns regarding infection control and the prevention measures that are applied to children in pediatric dental clinics in Saudi Arabia. The specific research question guiding this study was, “What are the perceptions and concerns of parents regarding infection control procedures and preventive measures implemented for their children during pediatric dental visits in Saudi Arabia?”

## MATERIALS AND METHODS 

### Study Design and Setting

This research utilized a descriptive, cross-sectional, electronic, questionnaire-based study design to assess parental perceptions and concerns regarding infection control measures in a pediatric dental setting post-COVID-19 pandemic. The study was carried out at the College of Dentistry at Qasim University and private dental clinics in the Buraydah region of Saudi Arabia from May 2025 to August 2025 using a convenience sampling technique.

### Sample Size and Study Population

The minimum sample size was calculated using the formula for cross-sectional studies with a setting assuming a 50% prevalence, a 5% margin of error, and a 95% confidence level. The required sample size was 385 participants. With consideration for potential incomplete responses or dropouts, the target enrollment was increased by 10% (423 participants). The target population included all parents visiting the dental clinics during the data collection period. The eligibility criteria required all parents to be aged 20 years or older with the native language of Arabic and be present for a normally scheduled appointment or walk-in. Parents under 20 years old, those who did not understand Arabic, parents working in the health sector, children visiting the clinic with relatives other than their parents, and parents who did not consent to the questionnaire or who left the survey incomplete were excluded.

### Data Collection Instruments and Procedures

The questionnaire used in this research is a modified version of one used in an earlier study.^16^ However, it should be noted that the sample population of the previous study consisted primarily of nonspecific dental patients, whereas the current research applied it to parents who are accompanying their children to the dental clinic. The content validity of the questionnaire was verified by an expert pedodontist to confirm consistency with the study objectives. A reliability assessment was conducted using psychometric testing (Cronbach’s alpha), which yielded a score of α = 0.71. Initially, the questionnaire was written in English; subsequently, it was translated into Arabic (the native language of the parent participants) and back-translated into English to ensure that the meaning of the original questionnaire had been preserved and to prepare for the final analysis. The questionnaire contains multiple items, including demographic characteristics (age, gender, and level of education), parental perceptions, and concerns regarding the use of essential PPE that was applied during their children’s dental visits (six closed-ended questions). The study utilized a self-reporting structure to gather the information from each participating parent (via an electronic device located in the reception area of each study location). For those participants who required assistance (such as illiterate parents), the receptionist provided a verbal explanation of the survey’s contents. Additionally, before completing the survey, the receptionist explained the study’s purpose, obtained verbal consent, narrated each question carefully, and explained all of the available responses to ensure the accuracy of the answers provided.

### Statistical Analysis 

All data were encoded and imported into SPSS v. 20.0 (IBM; Armonk, NY, USA) software. Descriptive analysis utilizing frequency and percentage was performed for qualitative data, and a chi-squared test was performed for the study. The comparisons were conducted based on the gender and educational level of the parents. A p-value < 0.05 was considered statistically significant.

### Ethical Considerations

This study was conducted according to the STROBE guidelines^24^ and in full accordance with the World Medical Association Declaration of Helsinki. The institutional review board at Qasim University (Buraydah, Saudi Arabia) approved the research project (No. 25-36-07). Furthermore, ahead of any data collection, written online consent with notification was obtained.

## RESULTS

### Demographic Characteristics of Parents

Based on the inclusion criteria, 400 parental responses were available for the final analysis. In terms of participant age, the majority of parents (43.2%) were in the 30-39 age group, and only 7.8% of the study population were in the 20-29 age group. Fathers accounted for 51.2% of the study population, while mothers accounted for 48.8%. Most parents (93.0%) had a university-level education, and only a small number of participants (0.3%) identified as illiterate. Table 1 details the demographic characteristics of parents.

**Table 1:** Demographic characteristics of the parent participants by the number of responses (N) and percentages (%)

Factors	Variables	N	%
Age groups in years	20-29	31	7.8
	30-39	173	43.2
	40-49	161	40.2
	≥ 50	35	8.8
Gender	Female	195	48.8
	Male	205	51.2
Level of education	Illiterate	1	0.3
	Primary school	5	1.2
	Secondary school	22	5.5
	University	372	93.0


### General Parental Response to the Questionnaire

Overall, the majority of parents believed that pediatric dentists should wear gloves (97.8%; 95% CI = 96.3, 99.2), dental masks (98%; 95% CI = 96.6, 99.4), and eyewear (89.5%; 95% CI = 86.5, 92.5) while treating their children. Furthermore, most of the parents believed it was essential to replace dental masks (76.3%; 95% CI = 72.1, 80.4) and clean eye goggles (79.0%; 95% CI = 75.0, 83.0) after treating each patient. Almost 50.5% (95% CI = 45.6, 55.4) of parents expressed no concern about contracting an infection at a pediatric dental appointment. Table 2 presents parental responses to the questionnaire for the whole sample with gender-specific comparisons.

**Table 2:** Parental questionnaire responses for the whole sample with gender-specific comparisons

Questions	Responses	Whole sample		Mothers(N = 195)	Fathers(N = 205)	Statistical analysis	
		N	% (95% CI)	N (%)	N (%)	Chi-squared	p-value
Do you think the pediatric dentist should routinely wear gloves while treating your child?	No	9	2.2 (0.8, 3.7)	4 (2.1)	5 (2.4)	1.829	0.0667
	Yes	391	97.8 (96.3, 99.2)	191 (97.9)	200 (97.6)		
Do you think the pediatric dentist should routinely wear a dental mask while treating your child?	No	8	2 (0.6, 3.4)	3 (1.5)	5 (2.4)	0.998	0.054*
	Yes	392	98 (96.6, 99.4)	192 (98.5)	200 (97.6)		
Do you think the pediatric dentist should routinely wear eye goggles while treating your child?	No	42	10.5 (7.5, 13.5)	23 (11.8)	19 (9.3)	2.804	0.042*
	Yes	358	89.5 (86.5, 92.5)	172 (88.2)	186 (90.7)		
How often do you think the pediatric dentist should change his/her mask?	After each patient	305	76.3 (72.1, 80.4)	150 (76.9)	155 (75.6)	1.58	0.048*
	When the pediatric dentist feels it is necessary	54	13.5 (10.2, 16.9)	30 (15.4)	24 (11.7)		
	Given visible contamination	41	10.3 (7.3, 13.2)	15 (7.7)	26 (12.7)		
How often do you think the pediatric dentist should be disinfecting his/her eye goggles?	After each patient	316	79 (75.0, 83.0)	154 (79.1)	162 (79.1)	2.229	0.079
	When the pediatric dentist feels it is necessary	52	13 (9.7, 16.3)	23 (11.8)	29 (14.1)		
	Given visible contamination	32	8 (5.3, 10.7)	18 (9.2)	14 (6.8)		
Are you concerned about an infection being transmitted to your child from the dentist/dental staff during a pediatric dentist visit?	Not concerned	202	50.5 (45.6, 55.4)	94 (48.2)	108 (52.7)	1.774	0.028*
	Somewhat concerned	184	46 (41.1, 50.9)	96 (49.2)	88 (42.9)		
	Very concerned	14	3.5 (1.7, 5.3)	5 (2.6)	9 (4.4)		
Statistical significance: p<0.05, chi-squared test . CI: confidence interval.							

### Parental Perceptions and Reported Behaviors

Table 3 presents parental responses with comparisons based on the respondent’s level of education. There were notable variations in parental responses to the closed-ended questions; however, comparisons were made both across gender and level of education. Statistically significant differences were noted in the parental responses, and the details are discussed in the following subsection.

**Table 3:** Parental responses according to educational level

Questions	Responses	Illiterate (N = 1)	Primary school (N = 5)	Secondary school (N = 22)	University (N = 372)	Statistical analysis	
		N (%)	N (%)	N (%)	N (%)	Chi-squared	p-value
Do you think the pediatric dentist should routinely wear gloves while treating your child?	No	0 (0.0)	0 (0.0)	3 (13.6)	6 (1.6)	1.277	0.042*
	Yes	1 (100.0)	5 (100.0)	19 (86.4)	366 (98.4)		
Do you think the pediatric dentist should routinely wear a dental mask while treating your child?	No	0 (0.0)	0 (0.0)	3 (13.6)	5 (1.3)	1.66	0.002*
	Yes	1 (100.0)	5 (100.0)	19 (86.4)	367 (98.7)		
Do you think the pediatric dentist should routinely wear eye goggles while treating your child?	No	0 (0.0)	0 (0.0)	9 (40.9)	33 (8.9)	2.227	0.022*
	Yes	1 (100.0)	5 (100.0)	13 (59.1)	339 (91.1)		
How often do you think the pediatric dentist should change his/her mask?	After each patient	1 (100.0)	5 (100.0)	11 (50)	288 (77.4)	2.648	0.037*
	When the pediatric dentist feels it is necessary	0 (0.0)	0 (0.0)	10 (45.5)	44 (11.8)		
	Given visible contamination	0 (0.0)	0 (0.0)	1 (4.5)	40 (10.8)		
How often do you think the pediatric dentist should be disinfecting his/her eye goggles?	After each patient	1 (100.0)	4 (80.0)	9 (40.9)	302 (81.2)	2.227	0.045*
	When the pediatric dentist feels it is necessary	0 (0.0)	0 (0.0)	10 (45.5)	42 (11.3)		
	Given visible contamination	0 (0.0)	1 (20.0)	3 (13.6)	28 (7.5)		
Are you concerned about an infection being transmitted to your child from the dentist/dental staff during a pediatric dentist visit?	Not concerned	0 (0.0)	1 (20.0)	10 (45.5)	191 (51.3)	3.555	0.030*
	Somewhat concerned	1 (100.0)	2 (40.0)	10 (45.5)	171 (46)		
	Very concerned	0 (0.0)	2 (40.0)	2 (9.1)	10 (2.7)		
p<0.05 is considered statistically significant (chi-squared test).							

#### Parental perceptions towards essential personal protective equipment (PPE)

**Gloves**

In response to the question, “Do you think the pediatric dentist should routinely wear gloves while treating your child?”, almost 97.8% of parents, whether mother or father, responded “yes”, while nearly 2.2% of parents responded “no”. These findings did not differ statistically significantly by parent gender (p = 0.0667). However, a statistically significant difference was noted for parent educational level (p = 0.042). The findings of the study indicated that most of the parents with a university education were unlikely to say “yes” (1.6%), while the great majority of the remaining participants agreed (98.4%). Furthermore, most parents with a secondary-school education almost always agreed (86.4%), while the others were unlikely to respond with “yes” (13.6%).

**Dental Masks**

In response to the question, “Do you think the pediatric dentist should routinely wear a dental mask while treating your child?”, most parents, regardless of their gender or education level, believed the pediatric dentist should routinely wear a dental mask (98%), while 2% of parents responded “no”, with a statistically significant difference noted between the genders (p = 0.054) and the parent education levels (p = 0.002). Regarding the parent genders, a majority of mothers (98.5%) and fathers (97.6%) answered “yes”. The percentage of those answering “no” was low for both genders, and it was slightly higher among fathers (2.4%) than mothers (1.5%). The comparison based on the level of education of parents indicated that most of the participants across all education levels, whether university (98.7%), primary school (100%), or illiterate (100%), answered “yes”. The group with secondary-school education showed markedly lower agreement, responding with “yes” (86.4%), while the remainder replied “no” (13.6%). This result means that parents with a secondary-school education were statistically significantly less likely to support wearing dental masks. In response to the question, “how often do you think a pediatric dentist should change their mask?”, 76.3% of the parents replied that the pediatric dentist should change the mask after each patient. The findings were statistically significant among the groups based on gender (p = 0.048) and level of parental education (p = 0.037). In a comparison of parent responses based on gender, most parents (76.9% mothers and 75.6% fathers) believed a pediatric dentist should change their mask after every patient. Opinions varied slightly on other options: more fathers (12.7%) than mothers (7.7%) selected “given visible contamination”. Regarding the comparison based on the parents’ educational levels, all parents who were illiterate or had a primary education (100%) selected “after each patient”. This option was the majority choice of parents with a university education (77.4%). However, only 50% of parents with a secondary-school education agreed, although it should be noted that 45.5% of parents with a secondary-school education reported that the mask should be changed when the pediatric dentist feels it is necessary.

**Eye Goggles**

In response to the question, “Do you think the pediatric dentist should routinely wear eye goggles while treating your child?”, 89.5% of parents, whether fathers or mothers, supported the routine use of eye goggles by the pediatric dentist; however, nearly 10.5% of parents responded “no.” A statistically significant difference was noted among parent genders (p = 0.042), with a slightly lower percentage of mothers (88.2%) agreeing compared to the fathers (90.7%). A greater statistically significant difference (p= 0.022) was observed among parental educational levels. Most university-educated parents (91.1%), along with 100% of the primary-educated and illiterate parents, supported the routine use of eye goggles. However, the secondary-education group exhibited a greater gap in support of the routine use of eye goggles (59.1%). This result means 40.9% disagreed, which indicates that parents with a secondary-school education were statistically significantly less supportive of the routine use of eye goggles than those with other educational levels. In response to the question, “How often should a pediatric dentist disinfect their eye goggles?”, 79% of parents believed the pediatric dentists should disinfect their eye goggles after each patient. No statistically significant difference was observed between parent genders (p = 0.079). The only statistically significant difference was noted between groups based on parental educational level (p = 0.045). Most parents across all levels of education answered that disinfection of the eye goggles should occur “after each patient” [illiterate (100%), primary-school education (80%), and university education (81.2%)]. However, this response was selected by only 40.9% of parents with a secondary-school education. Conversely, nearly half of parents with a secondary-school education (45.5%) believed that disinfection should only be performed when the dentist deems it necessary.

#### Parental concern regarding infection transfer 

In response to the question, “Are you concerned about an infection being transmitted to your child from the dentist/dental staff during a pediatric dentist visit,” 50.5% of parents responded that they were not concerned about the possibility of infection transmission during a pediatric dental visit. These findings show a statistically significant difference between the groups based on gender (p = 0.028) and levels of education (p = 0.030). The number of mothers who reported being somewhat concerned regarding the transmission of infection during pediatric dental appointments was 49.2% (compared to 42.9% of fathers), while 52.7% of fathers reported being not concerned (compared to 48.2% of mothers). Furthermore, the number of parents who were not concerned generally increased with higher education, while the number of very concerned parents markedly decreased: university-educated parents reported the highest level of “not concerned” (51.3%) and the lowest level of being “very concerned” (2.7%).

## DISCUSSION

Significant numbers of genes support human innate and adaptive immunity, which is crucial for surviving a hostile environment.^1^ As as consequence of children’s still-immature adaptive immunity, they are at a higher risk of infection with many pathogenic viruses, bacteria, fungi, and parasites.^20^ Previous research has demonstrated that racial or ethnic minority status, low household income, and family educational level are directly correlated with poor oral health in children, thereby increasing the risk of oral infection.^6^ It is evident that infection control strategies and protocols for preventing infection in children must be specifically designed and rigorously adhered to. The National Bureau of Economic Research reported that, following the implementation of enhanced infection control and prevention measures, child and infant mortality rates fell and continued to decline over the course of the 20th century.^7^ Most unintentional exposure to pathogenic microorganisms during dental treatment can be avoided if the dentist or healthcare providers strictly adhere to wearing essential PPE (gloves, dental masks, and protective eyewear) and carefully follow infection control protocols and guidelines. To reduce the acquired infections during dental procedures, it is important to follow the infection control guideline, take the proper precautions, follow the vaccination protocols, and adhere to infection control procedures following infection exposure.^14,19^

The Centers for Disease Control and Prevention (CDC) has recommended using PPE during all dental procedures and wearing protective eyewear by both the dentist and the patient, especially for those procedures that involve creating aerosols, spattering blood, or touching other body fluids.^12^ Furthermore, the parents are the primary caregivers for their children, and have the responsibility to ensure their child receives high-quality dental treatment in accordance with infection control protocols. Recognizing the importance of awareness regarding infection control measures among oral health care providers and parents warrants the strict observance of these measures. Furthermore, the present study indicated that most of the parents believed the pediatric dentist should routinely wear gloves (97.8%), a dental mask (98%), and eye goggles (89.5%) while treating their children. The results of the present study agree with previous studies,^5,16,18^ which demonstrated high levels of parental awareness toward PPE applied to their children during dental treatment.^5,18^ Another Saudi study focused on parents’ attitudes toward dental students wearing gloves, finding that 96% of parents emphasized the importance of wearing gloves during dental treatment.^2^ However, only 36.8% of participants in the Mousa et al^16^ study believed the dentist should wear “safety spectacles”. Furthermore, Bhayya and Shyagali^5^ reported that only 35% of parents preferred a pediatric dentist to wear a mask, and a similar percentage believed that a pediatric dentist should wear eye goggles. Moreover, in the present study, most of the parents (76.3%) believed that the pediatric dentist should change their dental mask after each patient, and 79% believed that they should disinfect their eye goggles after each patient. This result is inconsistent with the findings of Mousa et al,^16^ which reported that 29.1% and 27.8% of patients believed that dentists should replace their face mask or wash their “safety spectacles” if there is visible contamination.

If the right PPE is not worn during dental treatment, the risk of infection transmission is increased, so that parents become increasingly concerned about the health of their child. In the current study, 3.5% of parents were very concerned about the transmission of infection to their children during dental treatment, 46% were somewhat concerned, and 50.5% were unconcerned. However, these findings contradict the study by Mousa et al,^16^ which reported that 52.8% of patients were very concerned, 22.9% were somewhat concerned, and 24.3% were not concerned. In Saudi Arabia, dental clinics implement exceptionally high standards of infection control protocols, which explains why nearly half of the parents expressed no fear of their child contracting an infection during dental treatment. However, a study conducted by Kakarmath et al^11^ on the Indian population revealed that 76.4% of participants were positively aware of the increased risk of COVID-19 transmission during dental procedures, and 54.6% expressed apprehension about visiting dental clinics during the COVID-19 pandemic.

This study found that most Saudi Arabian parents reported positive perceptions regarding the use of PPE during their child’s treatment and demonstrated no concern about infection transmission during visits to pediatric dental clinics post-COVID-19 pandemic. However, two Saudi studies conducted by Farsi et al^8^ and Alhossine et al^3^ observed different attitudes toward pediatric dental visits during the COVID-19 epidemic. In the first of these studies, Farsi et al concluded that the mother would be unlikely to seek dental care unless in an emergency,^8^ while Alhossine et al reported that most parents only requested emergency treatment for their children in cases of severe suffering.^3^ Similarly, an Indian study revealed that 54.6% expressed apprehension of visiting dental clinics during the COVID-19 pandemic.^11^ The conclusion of the present study agrees with that of Nahir et al,^17^ who showed the level of parents’ knowledge and awareness was high concerning cross-infection control in pediatric dentistry during the COVID-19 pandemic. Similar findings were observed in numerous previous studies by Al Moherat and Aleissa,² Bhayya et al,⁵ and Sakarna et al.¹⁸ However, the findings of the present study showed greater improvements than those reported in the previous studies.^2,5,18^

In the present study, parents with a secondary-school education expressed less support for using PPE compared to those with higher educational levels. This finding may be attributed to several factors. First, health literacy likely plays an important role, as individuals with lower educational levels may have limited access to health information and obtain their information from unreliable, inaccurate sources, such as social media or personal networks. These sources could contribute to misconceptions regarding PPE. Second, previous experiences of dental visits may influence parents’ perceptions, and parents with lower educational levels may have less frequent dental visits or have different experiences that may shape their expectations and concerns towards infection control procedures. Third, cultural factors and social norms surrounding healthcare practices may vary across educational levels, potentially affecting perceptions and concerns about using protective measures such as gloves, dental masks, or eye goggles. Finally, socioeconomic factors often associated with educational levels, such as income and occupation, may indirectly influence parent perceptions by affecting access to healthcare information and quality of dental care experiences. The findings of this study have several important implications for improving parental awareness and adherence to infection control protocols in pediatric dental settings, highlighting the need to develop targeted educational campaigns that address gaps in knowledge among parents with lower education levels. These campaigns should utilize simple, accessible language and multiple formats, such as videos, brochures, and social media content, to ensure widespread understanding of the rationale behind infection control measures, and they should integrate infection-control education into school health programs. Additionally, the pediatric dentist needs to emphasize the importance of communicating about PPE with parents during dental visits and may also point out potential risks to their child. Furthermore, dental clinics could display posters or play educational videos in waiting areas to reinforce key messages about infection control in a non-threatening way. By implementing these recommendations, dental practices can not only improve parental adherence to infection control measures but also enhance the overall dental experience for children and their families, ultimately promoting better oral health outcomes.

It should be noted that the current study has several limitations: restricting the study to a specific region of Saudi Arabia and the use of a small study sample means that the sample does not represent the entire population of Saudi Arabia, which impacts the generalizability of the results. Additionally, the lack of studies concerning this area has made it challenging to make direct comparisons with the literature: most of the available studies focused on patients’ general perceptions of PPE. Therefore, pediatric studies, whether conducted before, during, or after the COVID-19 pandemic, are scarce. Furthermore, this study did not include socioeconomic indicators, which limits the ability to examine their potential association with participant perceptions. Future research should include socioeconomic variables to provide more comprehensive understanding. This, along with the findings from the current study can provide a valuable baseline for future pediatric research.

## CONCLUSIONS

This population of Saudi Arabian parents generally demonstrated positive perceptions regarding infection control measures in pediatric dental settings, and they were not concerned about contracting infections during dental visits. This finding confirms that clear infection control protocols are followed in Saudi Arabia. Furthermore, the findings indicate the need to supplement these protocols with accurate information to ensure adherence to them as well as guidelines in dental settings.

## ACKNOWLEDGMENT

The researcher would like to thank the Deanship of Graduate Studies and Scientific Research at Qassim University for financial support (QU-APC-2026).

## REFERENCES 
